# Setting Relationships between Structure and Devulcanization of Ground Tire Rubber and Their Effect on Self-Healing Elastomers

**DOI:** 10.3390/polym14010011

**Published:** 2021-12-21

**Authors:** Luis E. Alonso Pastor, Karina C. Núñez Carrero, Javier Araujo-Morera, Marianella Hernández Santana, José María Pastor

**Affiliations:** 1 Department of Condensed Matter Physics, University of Valladolid, Paseo del Cauce, 47010 Valladolid, Spain; luialo@cidaut.es (L.E.A.P.); jmpastor@fmc.uva.es (J.M.P.); 2 Foundation for Research and Development in Transport and Energy (CIDAUT), Parque Tecnológico de Boecillo, Plaza Vicente Aleixandre Campos 2, 47051 Valladolid, Spain; 3 Institute of Polymer Science and Technology (ICTP-CSIC), Juan de la Cierva 3, 28006 Madrid, Spain; jaraujo@ictp.csic.es

**Keywords:** end-of-life tires (ELTs), ground tire rubber (GTR), grinding process, devulcanization, self-healing

## Abstract

The use of devulcanized tire powder as an effective reinforcement in self-healing styrene-butadiene rubber (SBR) compounds has been investigated for the first time in this work. For this purpose, the evolution of the microstructure of the rubber from end-of-life tires (ELTs) was studied during granulation, grinding and devulcanization through an exhaustive characterization work in order to relate the final microstructure with the mechanical response of the repaired systems. Different morphologies (particle size distribution and specific surface area) obtained by cryogenic and water jet grinding processes, as well as different devulcanization techniques (thermo-mechanical, microwave, and thermo-chemical), were analyzed. The results demonstrated the key influence of the morphology of the ground tire rubber (GTR) on the obtained devulcanized products (dGTR). The predictions of the Horikx curves regarding the selectivity of the applied devulcanization processes were validated, thereby; a model of the microstructure of these materials was defined. This model made it possible to relate the morphology of GTR and dGTR with their activity as reinforcement in self-healing formulations. In this sense, higher specific surface area and percentage of free surface polymeric chains resulted in better mechanical performance and more effective healing. Such a strategy enabled an overall healing efficiency of more than 80% in terms of a real mechanical recovery (tensile strength and elongation at break), when adding 30 phr of dGTR. These results open a great opportunity to find the desired balance between the mechanical properties before and after self-repair, thus providing a high technological valorization to waste tires.

## 1. Introduction

Tires are mandatory products for the mobility of people and goods. However, these vital elements are not capable of being recycled due to their complex structures. Nowadays, the amount of waste tires discarded worldwide is approximately 800 million units, 10 million tons per year. If one considers that the amount of natural and synthetic rubber in tires is about 60%, 6 million tons of rubber from tires are disposed each year [1,2]. In this sense, giving a second life and valorization to end-of-life tires (ELTs) has become a global priority.

Some of the previously considered solutions for the disposal of ELTs (e.g., landfilling and incineration) have become less and less viable because of the associated environmental problems and high recovery costs. Instead, the reuse and valorization of the constituent materials have become the preferential solution. Methods and technologies that transform ELTs into raw materials primarily depend on the use of the tire (passenger cars, trucks, airplanes, etc.) since each application involves different rubber compounds. These technologies are grouped into three levels [3,4,5], following the circular economy model [6]. Level 1 includes the direct use of the tire, as well as mechanical treatments that destroy its structure (bead, sidewall or tread removal, cutting, compression, baling) [3,4,5]. Level 2 is made up of technologies that further reduce ELTs’ rubber size [3,4,5]. The resulting product, commonly called ground tire rubber (GTR), comes in different particle sizes in the form of chips (10–50 mm), granules (1–10 mm), and powder (<1 mm). There are three main technologies included in level 2: mechanical grinding, cryogenic grinding, and water jetting. Regarding GTR characteristics, there are significant differences between technologies. The GTR obtained with mechanical milling has a higher degree of oxidation and degradation caused by the large amount of heat generated during grinding; as the size is reduced, more damage is exerted to the rubber. Cryogenic GTR has a relatively smooth surface and a wide particle size distribution. GTR obtained with a water jet is porous and has a larger specific surface area compared to its cryoground peers [1]. In general, distinctions in the field of application are based on the particle size but not on the technology. The main uses of higher-size particles (chips and granules) include road and sport foundations, soil treatments, noise barriers, playground and sport surfaces, and footwear [4,7]. Meanwhile, powder is principally used as filler in rubberized asphalt [7,8,9,10], in building and construction and in concrete [7,9,11,12], as porous bitumen binders [4], in playground surfaces and trails for athletics [7,13] in automotive floor mats [7], and as filler for tires [4]. In addition, the outputs of level 2 are often used as feedstock for next-level processes. Level 3 mainly includes the pyrolysis and devulcanization [14] of ELT rubber. Pyrolysis refers to a thermal degradation in the absence of oxygen at a temperature range of 400–800 °C. The valuable products generated are pyrolysis gas, oil, and char (which consists of carbon black (CB), ashes, and inorganic particulates) [5]. Meanwhile, devulcanization is, by definition, the rupture of the sulfur–sulfur (S–S) and/or carbon–sulfur (C–S) bonds that constitute the three-dimensional structure formed during the vulcanization of the rubber [1,13,15]. However, this definition is somehow misleading, since it is commonly used to describe any process that aims to obtain a rubber compound that can be processed and vulcanized similarly to pristine rubber, regardless of whether the crosslinks or the main chain are broken. Currently, devulcanization is being approached by thermal, mechanical [16], chemical [17], biological, microwave [18], and ultrasonic techniques, as well as their combinations (e.g., thermo-chemical, thermo-mechanical, and mechano-chemical) [5,19,20,21]. Devulcanized products are very diverse and mainly distinguished by having different degrees of network breakage with the subsequent crosslink rupture and main chain scissions. Devulcanized GTR (dGTR) is primarily used to obtain thermoplastic elastomers (TPEs) [22], to obtain automotive parts, and as a secondary ingredient for tires [4]. However, the balance between network breakage and backbone chain scissions has an important effect on the final performance of the rubber product [1].

The main current challenge is related to improving the efficiency of the abovementioned technologies and to transforming ELTs into new raw materials and products with high added value. In line with this objective, research in which, for example, recycled fibers from car tire waste were used to successfully develop a rubber aerogel, has been carried out. Aerogels are ideal for many high-value applications such as drug-delivery pharmaceuticals, filters for pollutants, and building insulation materials [23,24,25]. It has also been discovered that tire pyrolysis produces value-added products such as tire pyrolysis oil (TPO) and char. TPO can be used as precursor in the synthesis of carbon nanotubes, and char can be converted to porous carbon structures that can be used as adsorption and energy-storage materials such as supercapacitors and batteries [26]. One previous work of the authors also contributed to the aforementioned objective, where GTR was used as effective reinforcement in self-healing materials [27]. In contrast to “classical” materials, self-healing materials are those capable of partially/completely restoring their initial properties and/or functionalities without significant human intervention [10]. There are basically two ways to develop self-healing polymers [28,29,30,31]. The first is based on the integration of discrete containers (capsules, fibers, or vascular networks) loaded with active components into the matrix material. When damage occurs, the containers break and release the healing agent to repair the damage. This is the so-called extrinsic concept. The second approach concerns the development of so-called intrinsically self-healing materials, that is, materials containing dynamic bonds that can restore their chemical or physical connections after damage under the influence of a non-disruptive external stimulus. A number of dynamic bonds have been shown to enable healing: Diels–Alder (DA) and retro-Diels–Alder (retro-DA) based bonds; hydrogen bonds in supramolecular networks; coordination complexes; disulfide-based chemistries; among others. However, these materials face a trade-off between mechanical performance and repairability—the higher the repairability, the lower the mechanical performance. One possible solution to overcome this trade-off is by reinforcing them. Hence, in this study, styrene-butadiene rubber (SBR) compounds that combine self-healing properties and good mechanical performance were developed thanks to the use of GTR as reinforcement. Contrary to analogue CB compounds, the mechanical performance of the self-healing rubber was enhanced (up to 80%) without adversely affecting the healing efficiency, resolving the problem of achieving both good reparability and good mechanical performance [27].

The work presented here is part of an ongoing research line that aims to develop elastomeric systems that can combine self-healing properties with the use of ELT rubber by acting on the tire rubber compound and using devulcanized rubber as reinforcement in self-healing matrices. We report the systematic and comparative microstructural analyses of diverse ground tire rubber (GTR) and devulcanized ground tire rubber (dGTR) originating from end-of-life truck tires (mainly composed of natural rubber (NR)) and obtained by different methods and technologies, thus enabling the definition of a structural model related to the selectivity of the applied devulcanization process. This is intended to demonstrates the possibilities of both the secondary raw material and the available recycling levels (i.e., level 2: grinding and level 3: devulcanization) used to valorize rubber from ELTs. Accordingly, self-healing SBR compounds filled with GTR and dGTR were prepared and characterized. These results were used to establish a relationship between the microstructures of GTR and dGTR and the self-healing efficiency and mechanical performance of SBR compounds. Finally, it is expected that this work will contribute to the perception of the recycling of ELTs as not only a benefit for the environment and the economy but also an opportunity for scientific-technological progress.

## 2. Materials and Methods

### 2.1. Materials

#### 2.1.1. Ground Tire Rubber (GTR)

Rubber granules from end-of-life truck tires were used. Two grinding technologies were further employed: cryogenic grinding and water jetting. Lehigh Technologies supplied cryogenic GTR (GTR-Cryo) and Rubber Jet supplied the GTR obtained with water jet technology (GTR-WJ), both within the same particle size range. Details on the particle size and composition of the as-received departure material (granules) and the resulting ground material (powder) are reported in Supplementary Material Table S1.

#### 2.1.2. Devulcanized Ground Tire Rubber (dGTR)

Three robust devulcanization techniques, based on different principles, were chosen to devulcanize the GTR powder samples. The procedures are described below.

##### Thermo-Mechanical (TM) Devulcanization

TM devulcanization was carried out in an internal mixer (Thermo Electron Corporation, Karlsruhe, Germany) with a filling volume of 70%, using Banbury-type rotors at room temperature and a rotor speed of 30 rpm for 10 min. Although the equipment was initially at room temperature, friction heated the material. As a result, the average process temperature was 70 °C.

##### Microwave (MW) Devulcanization

Microwave devulcanization was carried out in a microwave prototype [32,33]. Samples of 30 g were used. Devulcanization was performed at 700 W and 80 rpm/min, with an exposure time of 6 min.

##### Mechano-Chemical (CH) Devulcanization

Bis(3-triethoxysilyl propyl) tetrasulfide (TESPT) was used as devulcanizing agent following previously reported procedures [17,34,35]. GTR was extensively mixed with TESPT at a ratio of 6 mL of TESPT/100 g of GTR and subsequently soaked for 24 h prior to devulcanization. Then, the mixture was transferred to an internal mixer (Thermo Electron Corporation, Karlsruhe, Germany)) using Banbury-type rotors and a filling volume of 70%. The device was at room temperature at the beginning, the process time was 40 min, and the average temperature of the material was 67 °C.

#### 2.1.3. Self-Healing SBR Compounds

Styrene-butadiene rubber (E-SBR Europrene 1502) and commercial-grade vulcanizing additives supplied by Sigma-Aldrich (Burlington, MA, USA) were used as-received. Table 1 compiles all the prepared formulations.

Mixing was performed in an open two-roll mill (Comerio Ercole, Busto Arsizio, Italy) at room temperature using a rotor speed ratio of 1:1.5. First, rubber was passed through the rolls until a band was formed. The activating complex (zinc oxide (ZnO) and stearic acid (SA)) and filler (GTR or dGTR powder) were then progressively added to the rubber; finally, the curatives (N-cyclohexylbenzothiazole-2-sulphenamide (CBS) and sulfur (S)) were added.

The crosslinking process was followed using a Rubber Process Analyzer (Alpha Technologies, Bellingham, WA, USA) at curing temperature T_c_ = 160 °C, frequency of 0.833 Hz, and 2.79% strain for 60 min. The composites were then vulcanized in an electrically heated hydraulic press (Gumix, Fort Lee, NJ, USA) at 160 °C and 200 MPa according to their t_90_, as derived from the corresponding curing curves (see Supplementary Material Table S2). Samples were cut out from press-cured sheets to perform all the characterization and testing.

### 2.2. Characterization

#### 2.2.1. GTR and dGTR Characterization

##### Sol Fraction

About 5 g of GTR or dGTR were extracted (Soxhlet extraction) in acetone for 24 h and subsequently extracted in toluene for 72 h. After extraction, each sample was dried at 45 °C until a constant weight was reached. The sol fraction for each of the extractants was calculated with Equation (1).

(1) $$Sol\;\left(\%\right)=\left(1-\frac{W_{gel}}{W_{sample}}\right)\cdot 100$$

where $W_{gel}$ is the weight of the extracted sample and $W_{sample}$ is the weight of the sample before extraction.

The sol fraction was defined as the sum of the soluble fractions in acetone and toluene.

##### Crosslink Density

The crosslink density (*ν*) in the mass-basis form, which is the number of moles of sulfur crosslinks per unit mass of rubber, was determined through equilibrium swelling experiments. About 0.2 g of the acetone-extracted powder sample were placed in toluene at room temperature and in darkness—to avoid molecular changes—for 72 h. Analyzing samples in powder form without any additional processing avoids possible alterations of the crosslink density [33]. The toluene was refreshed every 24 h in order to ensure equilibrium swelling. The swollen samples were taken out from the solvent, carefully removing any solvent excess, and then weighed again. After that, samples were dried at 45 °C until a constant weight (~48 h).

Crosslink density was calculated using the Flory–Rehner equation [36] considering tetra-functional crosslinks; Equation (2). For further details, see Supplementary Material S3.

(2) v=−12·ρr·Vs· ln(1−Vr)+Vr+χ·Vr2Vr1/3−Vr2

##### Horikx Plots

M.M. Horikx derived a theoretical relationship between the soluble fraction generated after the degradation of a polymer network and the relative decrease in crosslink density as a result of either main-chain scission or crosslink breakage [37]. The application of the Horikx approach to evaluate devulcanization was experimentally verified by Verbruggen [38] and Seghar [39] in different ways. According to Horikx, when only main chain scission takes place, the relative decrease in crosslink density is given by Equation (3):

(3) $$1-\frac{v_{f}}{v_{i}}=1-\frac{{\left(1-\sqrt{s_{f}}\right)}^{2}}{{\left(1-\sqrt{s_{i}}\right)}^{2}}$$

where $v_{i}$ is the crosslink density of the untreated vulcanizate, $v_{f}$ is the crosslink density of the vulcanizate after treatment, $s_{i}$ is the soluble fraction of the untreated vulcanizate, and $s_{f}$ is the soluble fraction after treating the vulcanizate.

On the other hand, when only crosslink breakage takes place, the soluble fraction is related to the relative decrease in crosslink density by Equation (4):

(4) $$1-\frac{v_{f}}{v_{i}}=1-\frac{\gamma_{f}{\left(1-\sqrt{s_{f}}\right)}^{2}}{\gamma_{i}{\left(1-\sqrt{s_{i}}\right)}^{2}}$$

where the new parameters $\gamma_{i}$ and $\gamma_{f}$ are the average numbers of crosslinked units per chain before and after treatment, respectively.

Horikx plots are a representation of $s_{f}$ as a function of the relative decrease in the crosslink density, defined as:

(5) $$\mathrm{Relative}\;\mathrm{decrease}\;\mathrm{in}\;\mathrm{crosslink}\;\mathrm{density}=1-\frac{v_{f}}{v_{i}}$$

Horikx theoretical curves were drawn using Equations (3) and (4). For this, the initial soluble fraction $s_{i}$ was determined by the swelling test according to Equation (1), $s_{f}$ varied between $s_{i}$ and 1, and the crosslink indexes $\gamma_{i}$ and $\gamma_{f}$ were calculated from sol fraction by Equation (6), as described by Verbruggen et al. [38].

(6) s=(2+γ)−(γ2+4γ)122γ

The values of the treated vulcanizates were then plotted on the graph, and their positions with respect to the theoretical lines was evaluated.

The quantitative values of the network rupture and the selectivity of the devulcanization process could be estimated from the Horikx diagrams via the calculation of the percentage of devulcanization according to the methodology proposed by Edwards et al. [40], as detailed in Supplementary Material, S4.

##### Thermogravimetric Analysis (TGA)

Thermogravimetric curves were obtained using a thermal analyzer (Mettler Toledo, Columbus, OH, USA). Samples of ~10 mg were heated from 25 to 600 °C under a nitrogen atmosphere (inert medium) and in air (oxidant medium) from 600 to 1000 °C at a heating rate of 10 °C/min.

##### Scanning Electron Microscopy (SEM)

The morphological analysis of the GTR and dGTR powder and SBR compounds was achieved with scanning electron microscopy (Hitachi, Chiyoda, Tokyo, Japan). Samples were sputter-coated with gold–palladium prior to observation.

##### Particle Size Distribution

GTR and dGTR powder (~0.05 g) were previously dispersed in 20 mL of a water/ethanol 70/30 solution with 0.2 mL of the surfactant Triton X-100. The suspension was sonicated in an ultrasound bath (Elmasonic, Singen, Germany) for about 2 h. The particle size distribution was obtained by means of a laser scattering particle size distribution analyzer (Coulter, Barcelona, Spain). A volume-standard cumulative distribution was measured under stabilized conditions. Each sample was subjected to a 60 s optical measurement.

##### BET Surface Area

The BET surface areas of GTR powder were determined by nitrogen volume adsorption at −196 °C using a surface area and porosity analyzer (Micromeritics, Norcross, Georgia, USA). GTR was previously vacuum dried at 80 °C.

##### X-ray Photoelectron Spectroscopy

X-ray photoelectron measurements were performed on the surfaces of the GTR samples using a spectrometer (Fison Instruments, Ipswich, United Kingdom) equipped with a hemispherical electron analyzer (CLAM 2) and an Mg Kα X-ray source (1253.6 eV) operated at 300 W. Binding energies were corrected to the carbon 1s peak located at 285 eV.

##### Fourier Infrared Spectroscopy–Attenuated Total Reflectance (FTIR–ATR)

The FTIR–ATR spectra of dGTR and the products extracted with acetone and toluene were obtained using a Tensor 27 model Bunker spectrometer. The gel fractions were analyzed. Spectra were normalized to the SiO_2_ signal [33], and the relevant signals were analyzed [33,41] (see Supplementary Material Figure S5).

#### 2.2.2. Self-Healing Rubber Compounds Characterization

##### Tensile Testing

Dog-bone shape specimens (Type 2, UNE-ISO 37) were used for uniaxial tensile testing. Tests were done on a universal mechanical testing machine (Instron, Norwood, MA, USA) equipped with a 1 kN load cell. Samples were stretched until failure at a constant crosshead speed of 200 mm/min at room temperature. Stress at break (ultimate stress) and strain at break (ultimate strain) were determined in order to mechanically characterize the SBR compounds.

##### Healing Protocol

Dog-bone specimens were manually cut in the center with the aid of a razor blade, thus creating a proper joining area. In order to heal the specimens, the two separated parts were carefully repositioned together and fastened with clamps. Then, they were placed in an oven at 130 °C for 1 h. These conditions were selected as optimal after evaluating different healing protocols and using 70 °C and 7 h as departing conditions [42]. The thermally treated specimens were subjected to a tensile test with the abovementioned testing conditions. Healing efficiency (η) was calculated by Equation (7):

(7) $$\eta \left(\%\right)=\frac{P_{Healed}}{P_{Pristine}}\cdot 100$$

where $P_{Healed}$ and $P_{Pristine}$ are the property of interest (tensile strength or elongation at break) of the healed and pristine specimen, respectively, determined under the same test conditions.

## 3. Results and Discussion

The results derived from this research are divided in three sections. In the first section, we discuss the effect of the grinding technology (cryogrinding and water jet) on the microstructure of GTR. The second section is devoted to evaluating systematically various devulcanization techniques on different GTR samples and discussing their effectiveness towards selective devulcanization. In the last section, the incorporation of GTR and dGTR into a self-healing SBR compound is discussed, establishing proper relationships between the healing capability and the optimal and selective devulcanization technology.

### 3.1. Effect of Grinding Technology on the Microstructure of GTR

**Figure 1:**
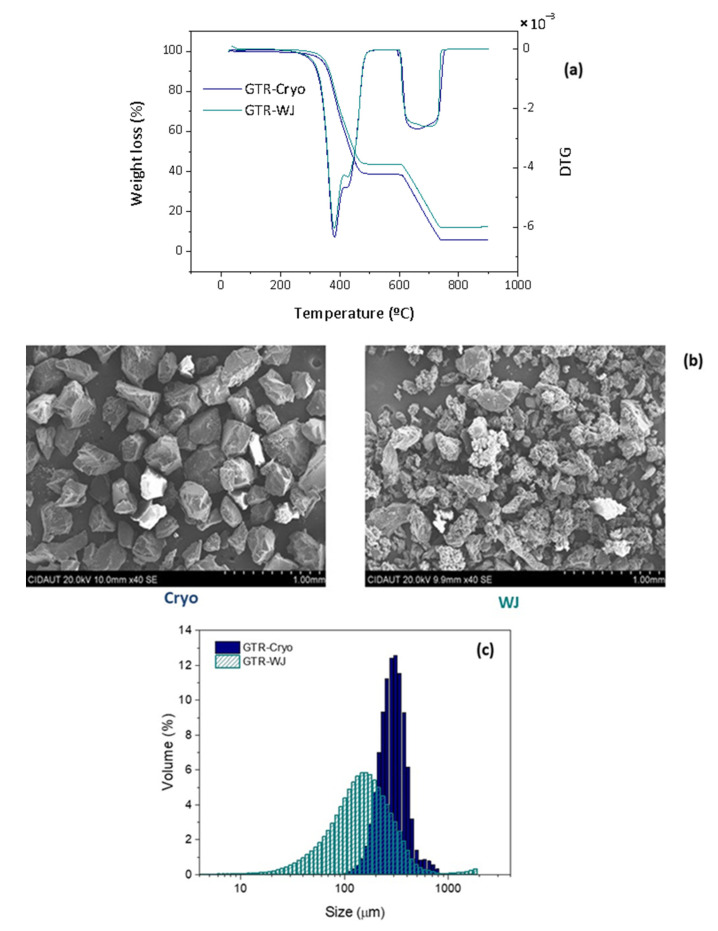
(**a**) DTG curves, (**b**) SEM micrographs, and (**c**) particle size distribution of the GTR obtained with cryogenic and water jet technologies.

TGA was conducted to study the effect of the grinding technology on the thermal stability of GTR and its composition (rubbers, fillers, ash, etc.). In Figure 1a, one can see three main losses that correspond to natural rubber (NR) (1st loss); a mixture of styrene-butadiene rubber (SBR), butadiene rubber (BR) (2nd loss), and carbon black (CB) (3rd loss), as previously reported by the authors [27]. It should be noted that GTR with a high NR content was purposely selected because this type of rubber more easily devulcanizes than SBR. When SBR is the major component of GTR, the network is more stable and less prone to devulcanize [1]. By looking at the first two losses, one can also notice that both industrial technologies (cryogrinding and water jet) did not seem to affect the degradation and relative content ratio of the resulting GTR powder. On the other hand, differences were observed in the third loss. A further analysis was carried out with XPS, which is discussed in Section 3.2.

Regarding the morphology of both GTR, SEM micrographs (Figure 1b) show important differences between the two technologies, as expected. The cryogenic technology seemed to produce bigger particles with a smooth surface; meanwhile, water jet powder seemed to be composed of more irregular particles with a broader size distribution. BET measurements and laser scattering measurements confirmed this observation, as seen in Figure 1c and Table 2. Other authors have found equivalent results [1,13,43].

**Figure 2:**
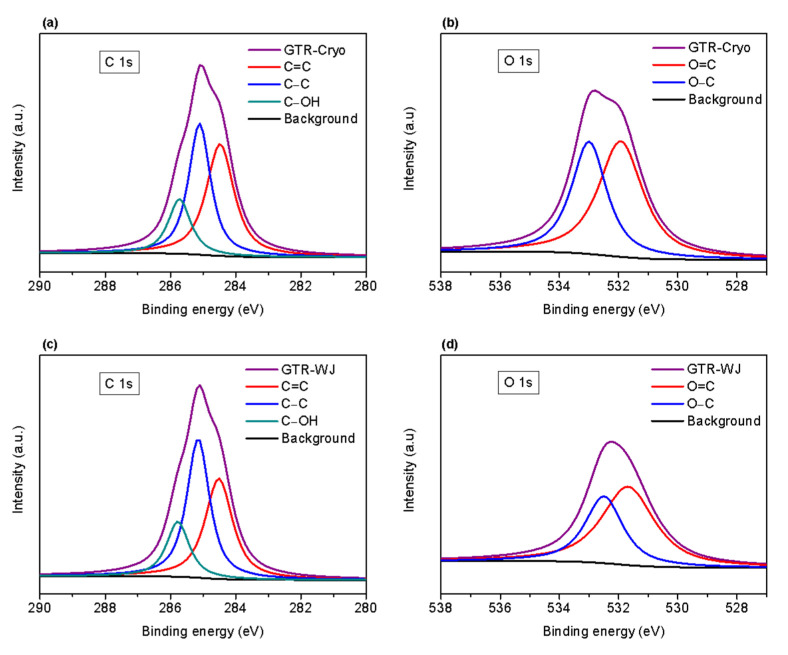
XPS C and O core spectra of: (**a**,**b**) GTR-Cryo and (**c**,**d**) GTR-WJ.

The chemical composition of the GTR surface was also investigated by means of XPS. The C 1s and O 1s core spectra of GTR-Cryo and GTR-WJ are shown in Figure 2. The deconvolution of the C 1s shows the characteristic peaks at binding energies of 284.5 eV (C=C), 285 eV (C–H), and 286.5 eV (C–OH) [6], with no noticeable differences between the two grinding methods. Meanwhile, the O 1s can be deconvoluted into two peaks related to double (O=C) and single (O–C) bonds at 530 and at 532.4 eV, respectively [44,45]. The intensity of both contributions seemed higher for GTR-Cryo, assuming that more oxygenated groups were present in this ground powder. The high compressive shear stress during the cryogrinding process could have generated active chains that could have subsequently been converted into oxidation products. Data in Table 2 show the relative element content (carbon (C), oxygen (O), and silicon (Si)) in each GTR. The higher O content in GTR-Cryo and the higher O/C ratio are evidence of the slight oxidation process occurring during the cryogrinding. As is discussed in the next sections, the morphology and structure of both GTR samples and their dispersion in the rubber matrix play decisive roles for achieving good mechanical and healing performance.

### 3.2. Comparison of Different Devulcanization Processes of GTR and Their Effect on Crosslink Breakage Selectivity

**Figure 3:**
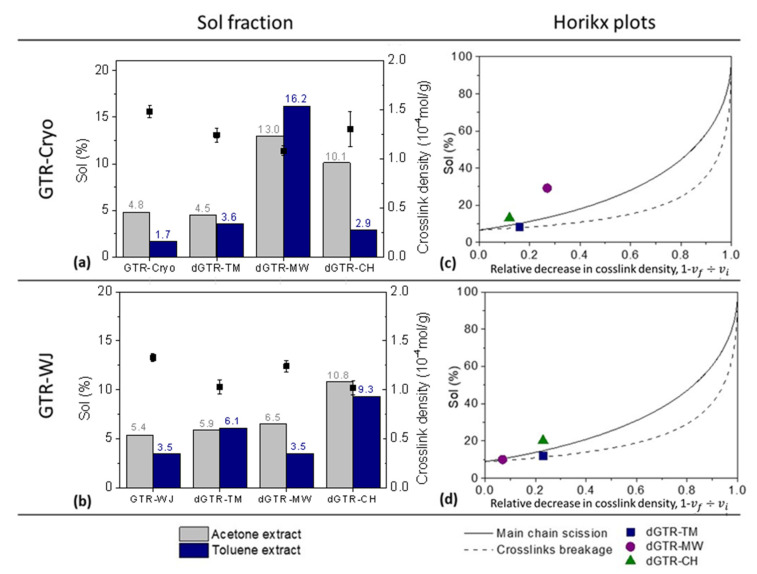
Sol fraction, crosslink density (**a**,**b**), and Horikx plots (**c**,**d**) of the GTR obtained with different grinding technologies and their devulcanized products.

Three devulcanization technologies based on different principles—thermo-mechanical (TM), microwave (MW), and mechano-chemical (CH)—were applied to the GTR studied in the previous section. Based on the trigger (temperature, shear forces, and radiation), it is possible to excite the atoms to enable the vibration of bonds and their rupture depending on the bond energy (S–S, 268 kJ/mol; C–S, 285 kJ/mol; C–C, 346 kJ/mol) [46]. Figure 3 shows a comparison of the properties of the devulcanized powder (dGTR) after applying the abovementioned techniques. Special attention has been paid to the soluble fractions extracted in both acetone and toluene, associated with free surface short and long chains, respectively (Standard ASTM D297-93), in order to correlate them with the resulting microstructures.

In the case of the dGTR obtained by the TM procedure, the Horikx plots show that regardless of the departing GTR (GTR-Cryo or GTR-WJ), the devulcanization was mostly selective (see Figure 3c,d). Both experimental points are positioned near the crosslink scission curve, meaning that the breakage of S–S bridges (with the lowest bond energy) dominated the reclamation of the rubber after being exposed to mechanical shearing. Moreover, the dGTR-TM obtained with GTR-WJ showed a lower crosslink density (1.03 vs. 1.24 × 10^−4^ mol/g) and a higher extracted fraction in toluene (6.1 vs. 3.6%) compared to its peer obtained with GTR-Cryo; additionally, a higher relative decrease in crosslink density was achieved for GTR-WJ. Hence, TM devulcanization seems more efficient when it is applied to WJ-derived particles. It is important to note that the departing GTR-WJ presented a higher percentage of extracted chains in toluene compared to GTR-Cryo (see Figure 3a,b). This higher sol fraction (understood as more free surface long polymeric chains [40,47,48]) plus the larger surface area and higher surface activity (due to the exposure to strong erosion from water jets) [43] of GTR-WJ contributed to the higher decrease in the network density.

The second devulcanization method analyzed was the MW. According to this technique, the composite interacts with the electromagnetic field, absorbs the MW radiation, and transforms it into heat to promote the breakage of main chain linkages and/or crosslinks [41]. Opposite to what was observed for TM devulcanization, significant differences were detected for the MW procedure depending on the characteristics of the departing GTR. For the dGTR-WJ particles, there were no significant changes in the toluene and acetone extractions or in the crosslink density compared with the baseline GTR-WJ. Meanwhile, the dGTR-MW obtained with GTR-Cryo showed noteworthy changes (↑270% and ↑950% in acetone and toluene extractions, respectively). The experimental point on the Horikx’s curves (Figure 3c) shows that these striking changes were mostly associated with main chain scission (degradation). Due to this degradation, high percentages of short polymeric chains could be extracted in acetone. In contrast, the point corresponding to the dGTR obtained with GTR-WJ (Figure 3d) demonstrates more selectivity towards devulcanization.

Some authors [33,49] have related the effectiveness of the MW technique to the composition of the sample, reporting that hydrophilic silica favors MW devulcanization. They suggested that the water absorbed by the silica evaporates when the ground powder is microwaved [49], acting as a catalyst and improving the devulcanization [33]. In this regard, it is not surprising that the dGTR obtained with GTR-Cryo (with its higher Si content related to the silica used as reinforcing filler, as evidenced by XPS) showed a higher relative decrease in crosslink density due to main chain scissions. Hence, one could conclude that the composition of waste rubber seems to have an important effect on the effectiveness of the subsequent MW devulcanization, while selectivity relies more on the previous grinding method.

Regarding the CH devulcanization technique, important differences were also observed between GTR-Cryo and GTR-WJ. A higher decrease in crosslink density was achieved in the dGTR obtained with GTR-WJ, in addition to a higher percentage of the toluene fraction. This fact could again be related to the higher specific surface area of GTR-WJ, which, in this particular case, enabled the better incorporation of the devulcanization agent [17,34,35] and thus higher efficiency in the reclaiming process. However, the results from the Horikx plots show that regardless of the used GTR, the CH devulcanization process was not selective and chain scission was predominant. These results contrast those obtained by Edwards et al. [40], who concluded that CH devulcanization is more selective for crosslink scission in comparison to TM devulcanization. A plausible explanation for this behavior could be related to the high percentages of acetone extract. We associate this extracted fraction with short, low-molecular-weight polymer chains [40,47,50], as well as with non-rubber components such as devulcanizing agents. Hence, the values of the sol fraction were higher than those traditionally reported based only on the soluble polymer contribution.

In brief, after the comparative analysis of three different devulcanization techniques, one can conclude that the microstructure of the departing GTR influences the obtainment of devulcanized products (dGTR), thus affecting the decrease in the network density and selectivity. Significant sensitivity was observed in the cases of MW and CH devulcanization due to the important influence of the shape and composition of GTR on these techniques. The TM technique showed less dependence on the GTR source. Likewise, it proved to be the most selective crosslink scission method studied according to the interpretation of the Horikx plots, which illustrated a slightly higher selectivity for dGTR-Cryo.

**Figure 4:**
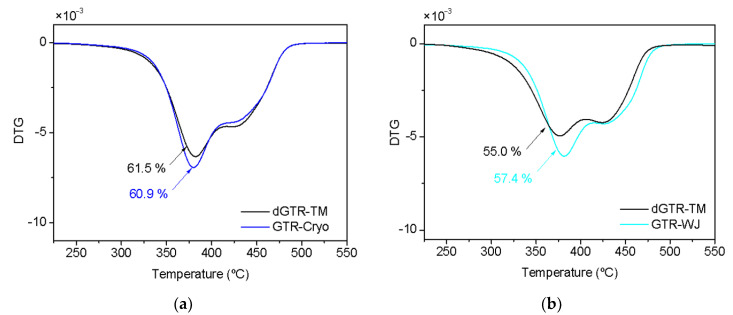
DTG curves of (**a**) GTR-Cryo, (**b**) GTR-WJ, and its dGTR-TM after extraction with acetone.

To demonstrate that correct readings of each component of the sol fraction and the Horikx curves were made, the monitoring of the weight loss via TGA was performed (Figure 4). One can see that there was no significant change in the content of degraded rubber fraction (composed of NR, SBR, and BR) between the departing GTR-Cryo/WJ and their devulcanized components (dGTR-TM) after extraction with acetone. This confirms that no substantial amount of short polymeric chains from degradation appeared after the TM devulcanization.

**Figure 5:**
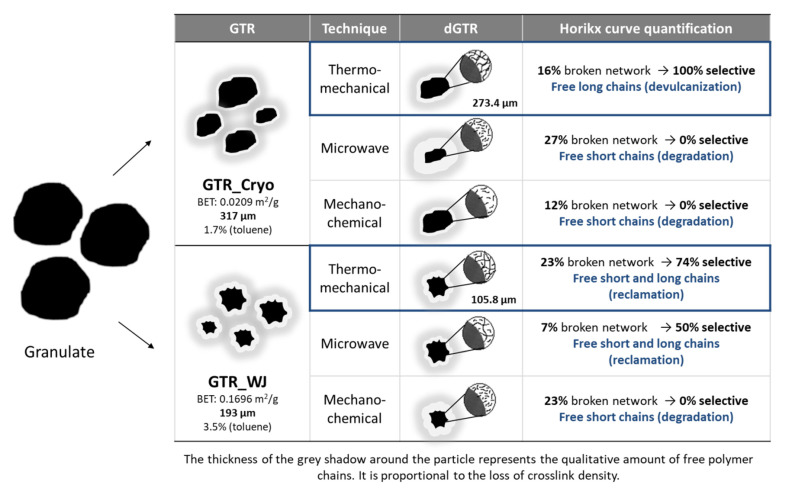
Representation of the microstructural evolution and main reclamation reactions.

With the obtained information and the quantitative analysis of the Horikx curves (see Supplementary Material, S4), a model of the evolution of the microstructure from granulated to devulcanized material has been proposed, identifying the predominant type of reclamation reaction (see Figure 5). The characteristics of the dGTR-TM devulcanization can be considered interesting for future applications as reinforcement thanks to the high percentages of achieved selectivity (100% and 74% for GTR-Cryo and GTR-WJ, respectively), although the percentages of devulcanization were relatively low (16% and 23%, respectively). This means that only the surface of GTR was devulcanized, thus obtaining a surface-devulcanized product [43]. It is important to highlight that the aim of this work was to obtain a “partially” devulcanized rubber that can act as reinforcement in self-healing matrices. This implies that the elastomeric behavior of GTR is needed. Thus, high devulcanization percentages would be inadequate for two reasons: (i) the non-devulcanized GTR fraction (which is the material that acts as reinforcement) would be too low, and the intended behavior would therefore not take place; (ii) in highly devulcanized samples, a large amount of CB would migrate into the self-healing matrix, which would significantly impair reparability [27].

From the point of view of the microstructure, the presence of free long chains on the surface of dGTR particles could be beneficial for improving their interactions with the components of the rubber formulation, favoring the formation of a good interphase and enabling co-crosslinking between dGTR and the rubber matrix while the tire rubber core acts as reinforcement. Additionally, the TM process analyzed here was conducted at room temperature, reducing energy costs and the generation of toxic and polluting substances. This condition, plus the fact that it does not involve the use of devulcanizing agents or solvents, provides an environmentally friendly and sustainable character to the TM devulcanization. In the next section, these benefits are explored for the first time in self-healing rubber compounds.

### 3.3. Correlating Microstructure and Devulcanization with Healing Capability of SBR Compounds

The final goal of this research was to propose a new valorized application to GTR and dGTR by incorporating them in a self-healing SBR compound. One key condition required to obtain healing is to have chain mobility through the damaged interface. This can be achieved by molecular interdiffusion/rearrangements and/or by the presence of dynamic bonds (chemical or physical reversible interactions) [51]. In the particular case of intrinsic healing, as in this research, the recovery is based on the reversibility of S–S bonds [10]. Thus, our aim was to correlate the previous microstructural model with the recovery of the mechanical properties of sulfur-cured SBR compounds after imposed localized macroscopic damage. Instead of a self-healing NR matrix (in accordance with the major component of the GTR used in this study), SBR was preferred due to its lower heterogeneity, as well as an extension of previous research [27]. In that study, we determined that SBR/GTR compounds can heal via disulfide exchange reactions. During the cryo-grinding of GTR, the scission of S–S crosslink bonds can occur, thus forming disulfide radicals that can combine with broken polymer chain radicals and recombine with other disulfide radicals.

**Figure 6:**
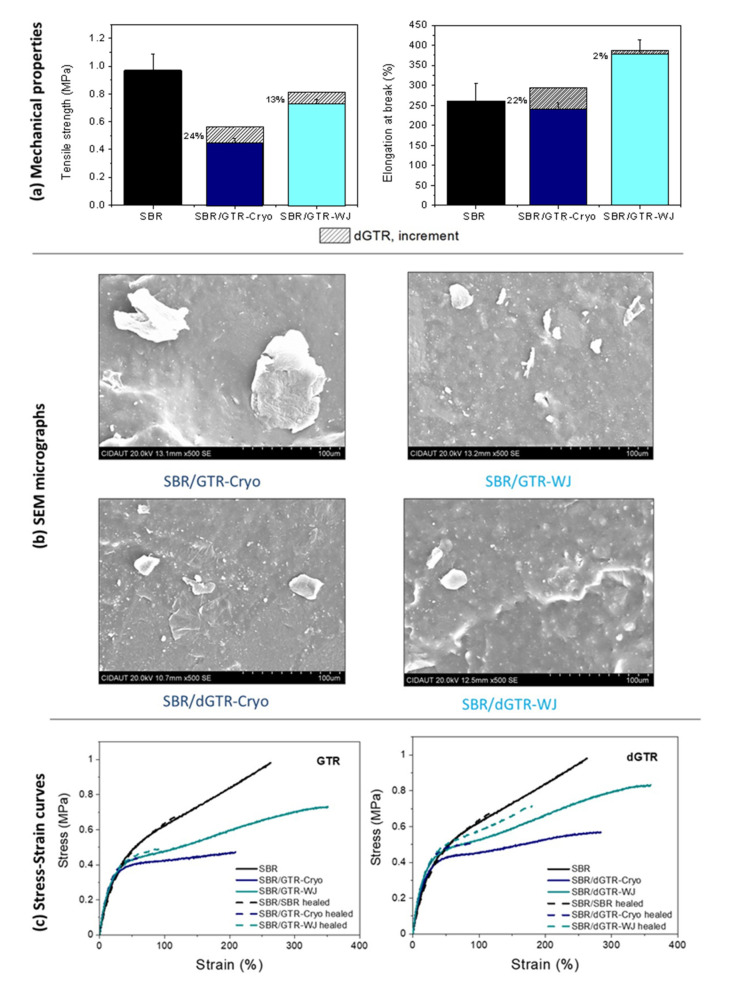
(**a**) Mechanical properties and (**b**) SEM micrographs of SBR/GTR and SBR/dGTR compounds; (**c**) tensile strength of self-healing SBR compounds with GTR and dGTR in the pristine (solid lines) and healed states (dashed lines).

We begin the discussion with the mechanical performance of pristine SBR compounds filled with 30 phr of GTR. The incorporation of both GTR (Cryo and WJ) decreased the mechanical properties of the SBR matrix (see Figure 6a). This behavior could be ascribed to two effects. On the one hand, the sulfur added to the SBR compound could have migrated from the matrix to GTR, reducing the concentration of S in the SBR/GTR compounds and, hence, their crosslink density and mechanical performance (see Supplementary Material Table S2). On the other hand, there seemed to be insufficient bonding between the particles and the virgin matrix. GTR particles were present as a dispersed phase consisting of large grains with poor compatibility and weak interactions with the SBR, as can be observed in SEM micrographs (Figure 6b) [13]. This weak interphase was improved by the use of smaller-sized devulcanized particles (see Supplementary Material Figure S6) that increased tensile strength, as shown in Figure 6a [52]. The elongation at break was also increased thanks to the plasticization effect of the higher sol fraction, as previously shown in Figure 3 [53]. Many authors have reported the use of compatibilizers (e.g., block and graft co-polymers) that act as bridges between GTR and polymeric matrices for reducing interfacial tension, thus achieving the finer dispersion of GTR in the matrix during blending and enabling morphology stabilization during processing and service life [54]. In this research, the devulcanized fraction was able to form a better interface between GTR and the SBR matrix through one of these mechanisms. One can also highlight the more obvious increases in tensile strength (24%) and elongation at break (22%) in the SBR/dGTR-Cryo when compared to SBR/dGTR-WJ. Thus, we can conclude that the 100% selectivity of the dGTR phase favors the mechanical performance of the SBR compounds.

The type of ground particles (Cryo or WJ) was also found to affect the mechanical behavior of the SBR compounds. As previously detailed, GTR-Cryo presented bigger particles with a smooth surface and smaller surface area. Additionally, the higher oxygen content on the surface (as determined from XPS data) favored hydrogen bonding and van der Waals interactions between the grains, resulting in a strong agglomeration tendency (see Figure 6b) [34]. Meanwhile, GTR-WJ presented smaller particles, the highest BET surface, and a higher sol fraction. As reported by many authors, interfacial adhesion directly increases with specific surface area [1,13] and the content of free polymeric chains [54]. Therefore, it is likely that the surface morphology of the WJ-derived particles explains the improved mechanical properties of the SBR/GTR-WJ compounds. Hence, the mechanical properties of the pristine SBR compounds seem to be very sensitive to both the shape of GTR and the content of free polymeric chains on its surface induced by the devulcanization process.

Figure 6c shows representative stress–strain curves for healed SBR compounds filled with GTR or dGTR. One can see that the healed SBR closely followed the behavior of the pristine SBR, fully recovering the instantaneous elastic modulus and the stress at low strains (<100%). The addition of a filler, either GTR or dGTR, showed an interesting and different effect. At low deformations, all the healed materials matched their pristine peers. However, as the tensile curves evolved, there seemed to be an improvement in the stress level, especially for the SBR/dGTR compounds, although the failure occurred at lower strains. A reasonable justification to this behavior could be as follows. Healing is a thermally triggered process. Temperature increases the mobility of free short and long chains, favoring the formation of entanglements. Additionally, some “revulcanization” of the residual sulfur that remains in GTR or dGTR enables the reformation of new S–S bridges. These two effects exerted a positive influence on the SBR compounds’ performance at the initial stages of deformation in this study. Similar results were reported elsewhere [52].

**Figure 7:**
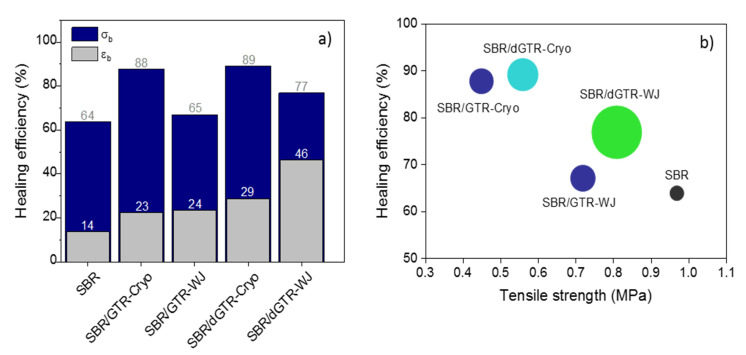
(**a**) Healing efficiency of SBR compounds with 30 phr of GTR/dGTR. (**b**) Overall healing performance of SBR compounds. The symbol size is properly scaled according to the recovery of the maximum strain.

Quantitatively, we determined the healing efficiency based on two parameters, the recovery of the elongation at break and of the tensile strength, as illustrated in Figure 7. The first reflection we can highlight is that, regardless of the type of particle (ground or devulcanized, Cryo or WJ), the recovery of the mechanical properties seemed to be higher than that observed for the unfilled SBR compound. This was expected. Mechanical properties and healing efficiency are antagonistic properties, especially when dealing with elastomeric materials. For healing to occur, chain mobility and dynamic bonds are required, and this opposes the formation of the irreversible covalent crosslinked network necessary to achieve good mechanical performance [51]. Hence, the decrease in tensile strength shown by the filled compounds resulted in higher healing capabilities. This divergent effect is also valid for explaining the almost full recovery (healing efficiency of 88%) of the SBR compound filled with 30 phr of GTR-Cryo compared to its equivalent with 30 phr of GTR-WJ (healing efficiency of 65%, similar to that of the pure SBR).

The second aspect worth analyzing is the effect of the devulcanization on the healing capability of SBR compounds. From Figure 7a, one can confirm that such a process favored healing in this study. During the TM devulcanization, the selective homolytic scission of S–S took place, thus enabling viscous flow and enhancing the mobility of free short and long polymer chains. Consequently, the interdiffusion of rubber chains and the rearrangement of broken, reversible S–S bonds at the healed interface were favored. If we correlate this behavior with the structural model, one can state that the more selectively devulcanized the material (dGTR-Cryo), the higher mechanical recovery one can achieve in terms of tensile strength. Meanwhile, if we analyze the recovery in terms of elongation at break, the dGTR-WJ showed the highest value. Tensile testing is a standard technique for the determination of self-healing efficiency [55]. The most commonly used parameter is the maximum load at failure of the specimen (tensile strength). However, healing in terms of maximum deformation is of paramount importance in the field of elastomeric materials. The recovery beyond low strains indicates that significant load transfer from the damaged/repaired zone to the bulk occurred, leading to higher deformation before failure. In this sense, the SBR/dGTR-WJ compound seems to be the principal choice showing the best overall healing performance in terms of a real mechanical recovery (stress and strain). Figure 7b illustrates a very good representation of these results.

## 4. Conclusions

This study explored the effect of free polymeric chains on the surface of partially devulcanized recycled tire powder (from end-of-life truck tires) in self-healing SBR compounds. The importance of understanding the evolution of the microstructure during the different levels of the recycling of ELTs (i.e., grinding (GTR) and devulcanization (dGTR)) has also been demonstrated via the establishment of relationships between particle shape, composition, and specific surface area with the efficiency of various devulcanization processes. First, we found that the microstructure of the departing GTR influenced the obtainment of devulcanized products (dGTR), affecting the decrease in the network density and selectivity. Secondly, the thermo-mechanical devulcanization (TM) provided the highest amount of free long chains on the surface of the ground powder, regardless of the type of departing grinding method (cryogenic or WJ). This devulcanization technique also proved to be the most selective crosslink scission method when dealing with end-of-life truck tires.

A selected content (30 phr) of GTR and TM-dGTR particles was further added to a self-healing SBR compound. The morphological and dynamic features of the grains suggested that the key factors leading to the recovery of the mechanical properties are the high percentage of free surface polymeric chains and high devulcanization selectivity, achieving a healing efficiency of more than 80% based on the recovery of both stress and strain. In conclusion, the research discussed here gathered information at different levels (molecular and micro/macro), and it presents a good phenomenological approach for a better understanding of the underlying healing mechanism taking place in elastomeric materials. Future work will deal with the optimization of devulcanized particle content, as well as the incorporation of GTR/dGTR in filled SBR compounds, in the search of better mechanical properties to be scaled up to real-life applications.

## Figures and Tables

**Table 1:** SBR compounds recipes in phr (parts per hundred parts of rubber).

Ingredient (phr)	Compound				
	SBR	SBR/GTR-Cryo	SBR/GTR-WJ	SBR/dGTR-Cryo	SBR/dGTR-WJ
SBR	100	100	100	100	100
ZnO	5	5	5	5	5
SA	1	1	1	1	1
CBS	1	1	1	1	1
S	1	1	1	1	1
GTR		30	30		
dGTR				30	30

**Table 2:** Average particle size, BET surface, and surface relative element content of the GTR obtained with cryogenic and water jet technologies.

	Powder Sample	
	GTR-Cryo	GTR-WJ
Average particle size (μm)	317 (6)	193 (33)
BET surface area (m^2^/g)	0.0209	0.1696
Element content (%)		
C	82.37	88
O	13.59	10.67
Si	4.04	1.33
O/C	0.16	0.12

## Data Availability

The data that support the findings of this study are available on request from the corresponding author, M.H.S.

## Supplementary Materials

The following are available online at https://www.mdpi.com/article/10.3390/polym14010011/s1. Table S1: Composition and particle size of as-received rubber granules and GTR powder, reported by the supplier; Table S2: Data derived from the curing curves and crosslink density of SBR compounds; S3: Crosslink density, S4: Quantification of selectivity parameter from Horikx plots, Figure S5. (a) FTIR-ATR spectra of dGTR_WJ-TM and the extracted products (gel fractions). Zoom on the regions of interest: (b) C–H and (c) C=C signals, Figure S6. Particle size distribution of GTR and dGTR from cryogrinding and water jet technologies.
