# Supplementary material for: Setting Relationships between Structure and Devulcanization of Ground Tire Rubber and Their Effect on Self-Healing Elastomers
Source: Polymers (Basel). 2021 Dec 21;14(1):11. doi: 10.3390/polym14010011 (PMC8747371; doi:10.3390/polym14010011)
Supplement: Supplementary file 1 [file polymers-14-00011-s001.zip › polymers-1494684-supplementary.pdf]

polymers

MDPI

*Supplementary Materials*

# Setting Relationships between Structure and Devulcanization of Ground Tire Rubber and Their Effect on Self-Healing Elastomers

**Luis E. Alonso Pastor [1], Karina C. Núñez Carrero [2,*], Javier Araujo-Morera [3], Marianella Hernández Santana [3,*] and José María Pastor [1,2]**

[1] Department of Condensed Matter Physics, University of Valladolid, Paseo del Cauce, 47010 Valladolid, Spain; luialo@cidaut.es (L.E.A.P.); jmpastor@fmc.uva.es (J.M.P.)
[2] Foundation for Research and Development in Transport and Energy (CIDAUT), Parque Tecnológico de Boecillo, Plaza Vicente Aleixandre Campos 2, 47051 Valladolid, Spain
[3] Institute of Polymer Science and Technology (ICTP-CSIC), Juan de la Cierva 3, 28006 Madrid, Spain; jaraujo@ictp.csic.es
* Correspondence: karnun@cidaut.es (K.C.N.C.); marherna@ictp.csic.es (M.H.S.)

**Table S1.** Composition and particle size of as-received rubber granules and GTR powder, reported by the supplier.

| | Granules | | Powder | |
|---|---|---|---|---|
| | **Cryo** | **WJ** | **GTR_Cryo** | **GTR_WJ** |
| Polymer content (%) | * | 59.0 | 57.3 | 57.4 |
| CB content (%) | * | 30.7 | 30.7 | 29.9 |
| Particle size (mm) | 4 - 2 | 4 – 3 | < 0.4 | < 0.4 |

* not reported.

**Table S2.** Data derived from the curing curves and crosslink density of SBR compounds.

| **Compounds** | **ML (dNM)** | **MH (dNM)** | **ΔM (dNM)** | **$t_{s2}$ (min)** | **$t_{90}$ (min)** | **ν ($10^{-4}$ mol/g)** |
|---|---|---|---|---|---|---|
| SBR | 0.7 | 5.8 | 5.1 | 17 | 27 | 0.32 (0.01) |
| SBR/GTR-Cryo | 0.9 | 3.2 | 2.3 | 21 | 24 | 0.09 (0.01) |
| SBR/GTR-WJ | 0.8 | 3.6 | 2.8 | 11 | 19 | 0.09 (0.01) |
| SBR/dGTR-Cryo | 1.1 | 3.3 | 2.2 | 23 | 26 | 0.08 (0.01) |
| SBR/dGTR-WJ | 0.9 | 3.7 | 2.7 | 11 | 19 | 0.06 (0.01) |

### S3. Crosslink density

Crosslink density was calculated using the Flory–Rehner equation (Flory et al., 1943) considering tetra-functional crosslinks, equation (1):

$$\boldsymbol{v} = -\frac{1}{2 \cdot \rho_r \cdot V_s} \cdot \frac{\ln(1 - V_r) + \boldsymbol{V_r} + \boldsymbol{\chi} \cdot \boldsymbol{V_r}^2}{\boldsymbol{V_r}^{1/3} - {}^{V_r}/_{2}} \quad (1)$$

where $v$ is the crosslink density per unit mass of the polymer network (mol/g), $\chi$ is the Flory–Huggins interaction parameter (0.39 for toluene and natural rubber (NR), since NR is the major rubber component in truck tires), $\rho_r$ is the density of the rubber (determined by hydrostatic weighing method in accordance with the standard ISO 2781) and $V_s$ is the molar volume of toluene (106.2 cm$^3$/mol). $V_r$ is the apparent volume fraction of the polymer in the swollen gel phase of the sample, calculated according to equation (2) Bilgili et al., 2001 and Colom et al., 2018):

$$V_r = \frac{W_d/\rho_r}{W_d/\rho_r + W_t/\rho_s} \quad (2)$$

where $W_d$ and $W_t$ are the weight of the dried and toluene swollen sample, respectively. $W_t$ is calculated as the weight difference between the sample in the swollen and dry state. Finally, $\rho_s$ is the density of the swelling solvent (0.87 g/cm$^3$ for toluene).

**S4. Quantification of selectivity parameter from Horikx plots**

Edwards et al (Edwards et al., 2016) considered two possible approaches: defining the selectivity parameter in the sol fraction (vertical) direction or in the crosslink density (horizontal) direction, as shown in the figure below.

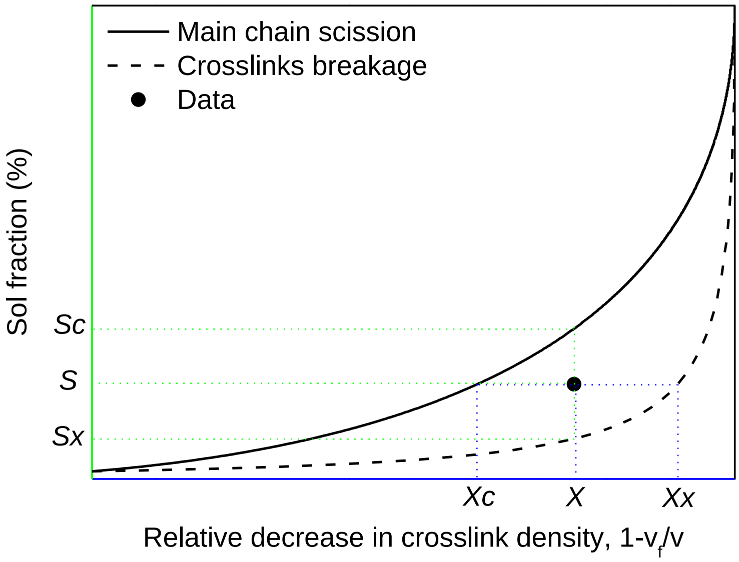


The selectivity (K) can be quantitatively determined through the expression (3):

$$K = \frac{K_s + K_x}{2} \quad (3)$$

where $K_s$ and $K_x$ are defined according to eq. (4) and (5) respectively:

$$K_s = \frac{S_c - s}{S_c - S_x} \quad (4)$$

$$K_x = \frac{X - X_c}{X_x - X_c} \quad (5)$$

If devulcanization is defined as the decrease of the network density due to the selective breakage of crosslinks, both selectivity and network density must be considered. Thus, the devulcanization percentage can be calculated through eq. 6:

$$Devulcanization\ (\%) = \left(\frac{Selectiviy + Relative\ decrease\ in\ crosslink\ density}{2}\right) \times 100 \quad (6)$$

| | Cryo | | | WJ | | |
|---|---|---|---|---|---|---|
| | **TM** | **MW** | **CH** | **TM** | **MW** | **CH** |
| **Selectivity (%)** | 100 | 0 | 0 | 74 | 50 | 0 |

**S5. FTIR of gel fractions**

Figure 1 shows FTIR-ATR spectra of dGTR_WJ-TM and the products extracted with acetone and toluene (gel fractions). In the Figure 1b it can clearly be seen how after the extraction with acetone (dGTR_WJ-TM-Acet) the C-H signal, between 3000 and 2800 $cm^{-1}$, decrease its intensity. Taking into account the bibliography (Colom et al., 2016 and; Aoudia et al., 2017), this can be related with the extraction of plasticizers or oils. Regarding the sample extracted firstly with acetone and then with toluene (dGTR_WJ-TM-Tol), a decrease in the signal of both C-H and C=C, signal at 1660 $cm^{-1}$, is observed; see Figure 1c. Consistent with the literature (Colom et al., 2016 and; Aoudia et al., 2017), the decrease in both signals can be attributed to the loss of free NR and SBR chains.

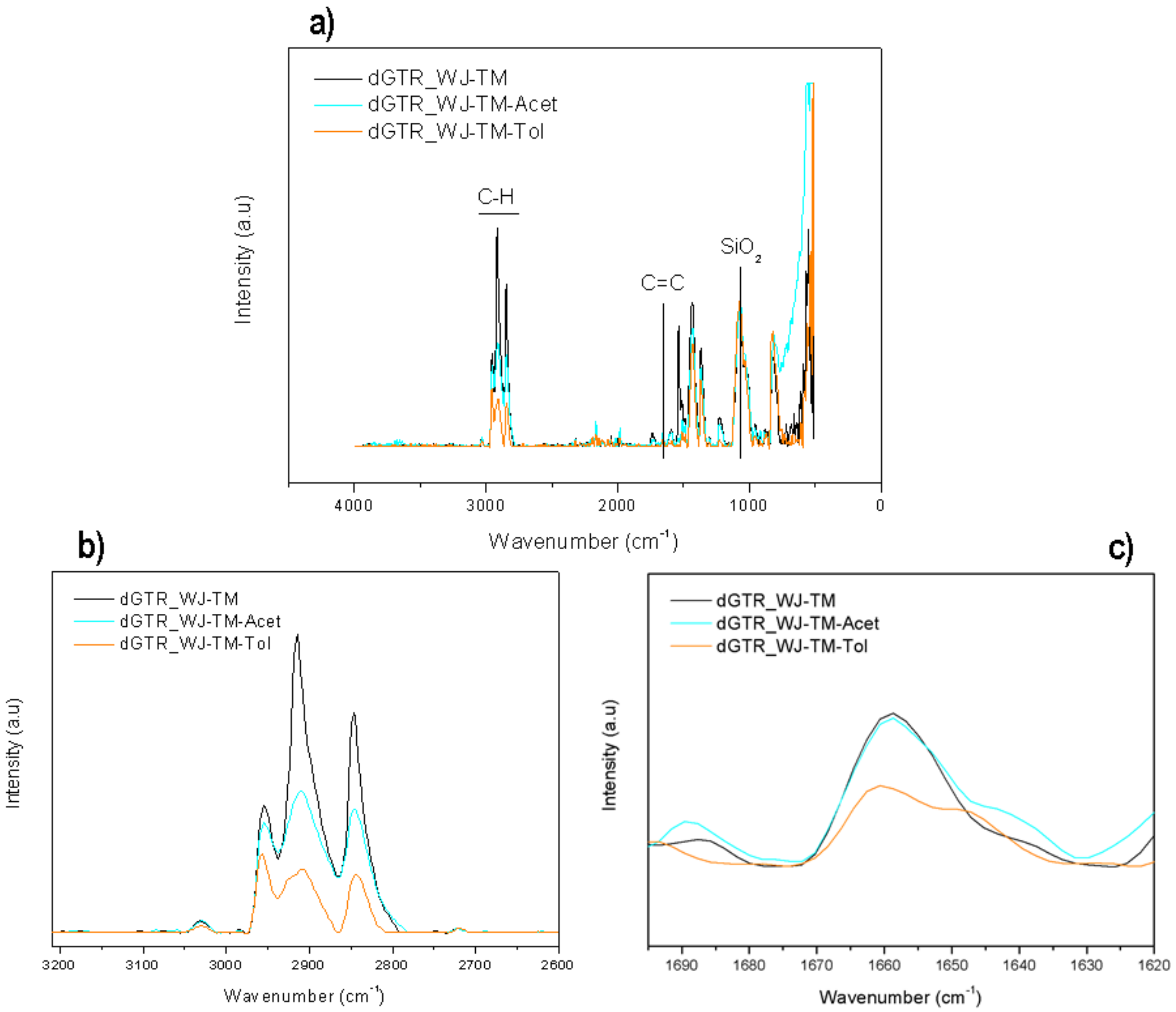


**Figure S5.** (**a**) FTIR-ATR spectra of dGTR_WJ-TM and the extracted products (gel fractions). Zoom on the regions of interest: (**b**) C–H and (**c**) C=C signals.

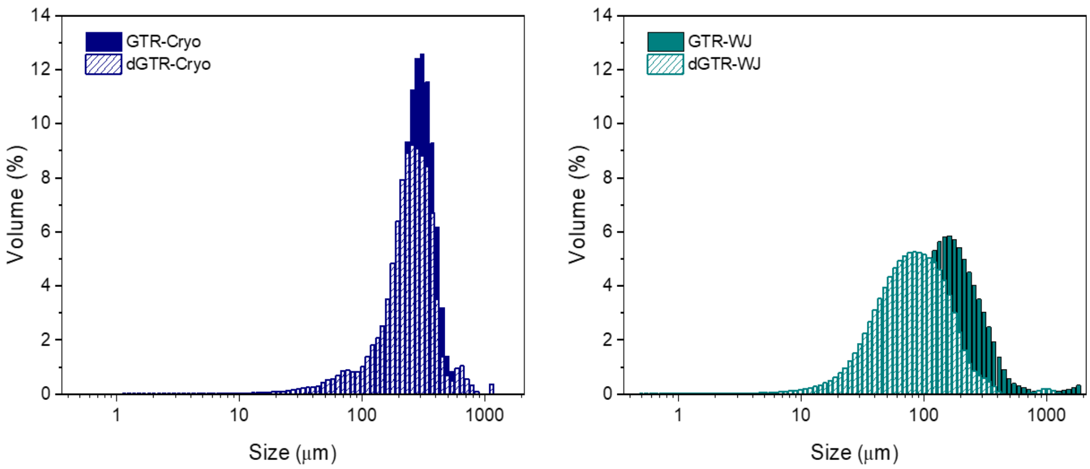


**Figure S6.** Particle size distribution of GTR and dGTR from cryogrinding and water jet technologies.
